# Genome sequencing of bacteria: sequencing, *de novo* assembly and rapid analysis using open source tools

**DOI:** 10.1186/1471-2164-14-211

**Published:** 2013-04-01

**Authors:** Veljo Kisand, Teresa Lettieri

**Affiliations:** 1 Institute of Technology, Tartu University, Nooruse 1, Tartu 50411, Estonia; 2 European Commission, Joint Research Centre, Institute for Environment and Sustainability Rural, Water and Ecosystem Resources Unit, TP 270, Via E. Fermi, 2749, Ispra, VA, 21027, Italy

**Keywords:** Reference mapping, *De novo* sequencing, *De novo* assembly, Automated annotation, Marine bacteria

## Abstract

**Background:**

*De novo* genome sequencing of previously uncharacterized microorganisms has the potential to open up new frontiers in microbial genomics by providing insight into both functional capabilities and biodiversity. Until recently, Roche 454 pyrosequencing was the NGS method of choice for *de novo* assembly because it generates hundreds of thousands of long reads (<450 bps), which are presumed to aid in the analysis of uncharacterized genomes. The array of tools for processing NGS data are increasingly free and open source and are often adopted for both their high quality and role in promoting academic freedom.

**Results:**

The error rate of pyrosequencing the *Alcanivorax borkumensis* genome was such that thousands of insertions and deletions were artificially introduced into the finished genome. Despite a high coverage (~30 fold), it did not allow the reference genome to be fully mapped. Reads from regions with errors had low quality, low coverage, or were missing. The main defect of the reference mapping was the introduction of artificial indels into contigs through lower than 100% consensus and distracting gene calling due to artificial stop codons. No assembler was able to perform *de novo* assembly comparable to reference mapping. Automated annotation tools performed similarly on reference mapped and *de novo* draft genomes, and annotated most CDSs in the *de novo* assembled draft genomes.

**Conclusions:**

Free and open source software (FOSS) tools for assembly and annotation of NGS data are being developed rapidly to provide accurate results with less computational effort. Usability is not high priority and these tools currently do not allow the data to be processed without manual intervention. Despite this, genome assemblers now readily assemble medium short reads into long contigs (>97-98% genome coverage). A notable gap in pyrosequencing technology is the quality of base pair calling and conflicting base pairs between single reads at the same nucleotide position. Regardless, using draft whole genomes that are not finished and remain fragmented into tens of contigs allows one to characterize unknown bacteria with modest effort.

## Background

Whole Genome Shotgun (WGS) sequencing of novel environmental bacterial isolates or single amplified genomes (SAGs) of uncultured bacteria using Next-Generation Sequencing (NGS) technologies opens up new perspectives in microbial ecology studies. Species with defined genomes increase the value of many different biological studies, e.g. to compare the roles of organisms in the environment at different levels - microbial evolution, metabolism, and ecology [1]. Fully sequenced bacterial genomes are superior to genome fragments because they provide the only accurate reference for interpreting meta-genomes and -transcriptomes. Relatively inexpensive NGS technologies can produce large quantities of sequencing data, but most NGS methods produce relatively short sequence fragments i.e. short read sequences (SRSs, shorter compared to Sanger reads). These SRSs need to be assembled, perhaps into full genomes. Bacterial genome assembly is a computational process in which SRSs are compiled into whole genome sequences; in the case of *de novo* assembly no preliminary information about the genome is available, while reference mapping uses existing reference genome sequences to assemble SRSs from re-sequencing projects.

Recent advances in next-generation sequencing (NGS) technologies have moved whole genome sequencing from large centres to small research groups and even individual scientists. This move is largely fuelled by a reduction in sequencing costs. However, what is lacking is a set of standardized analysis tools that can be used by non-bioinformaticians. There are millions of uncharacterised environmental microbes that lack close relatives with finished and annotated genomes, and several essential computational problems need to be addressed before the information contained within these genomes can be fully accessed. Several algorithms have been developed to assemble short (<100 bp) and medium reads (e.g. the Roche 454 FLX Titanium platform with average read length of ~400 bp was released in October 2008), including efforts by the commercial providers of sequencing technologies (e.g. the Newbler assembler from Roche). Currently, [2], the Roche 454 FLX Titanium provides up to a 1 kb read length, which is comparable to Sanger sequencing, so the coverage needed to assemble bacterial genomes will decrease along with the computational power required per bacterial genome. An emerging and competing technology (since late 2012) that is able to produce ~400 bp read via paired end sequencing is the 2×250bps by Illumina MiSeq.

Assembly is followed by gene prediction/annotation, a computational process in which regions of the DNA containing coding genes are identified. Advanced gene annotators typically use complex probabilistic models such as hidden Markov models (HMMs in HMMer [3]) or BLAST [4]. HMMs need to be trained, a process that depends on existing information, i.e. on genomes that are already annotated, so genes with very different nucleotide compositions may be missed using this approach. Second-generation annotation tools combine various gene prediction algorithms, which ought to increase their accuracy in performing gene annotations [5].

It is clear that existing tools for sequence annotation involve extensive manual work which is currently not feasible for coping with the increasing amounts of genome data from numerous species simultaneously. However, the use of fully automated annotation tools may lead to error propagation and biologically irrelevant data. To date, there has been no exhaustive evaluation of genome analysis pipelines from assembly to annotation (see [6]). To complicate such an evaluation there is an increasing variety of online and locally-running pipelines ([6] and references therein). Probably the best-known online automated annotation systems are RAST (Rapid Annotation using Subsystem Technology), IMG (Integrated Microbial Genomes [7]), JCVI annotator (J. Craig Venter Institute), PGAAP (Prokaryotic Genomes Automatic Annotation Pipeline at NCBI [8]), and RaTT [9]. The RAST system [10], which is integrated within the SEED environment [11], allows one to browse the annotated genomes and supports the use of external comparative tools to analyse them. Gene calling in IMG ([12] and references therein) is based on BLASTp, after which genes are assigned using various annotation databases such COG, Pfam, TIGRfam and Gene ontology. Functional annotations can be characterized by COG functional categories, KEGG and MetaCyc pathway collections. NCBI PGAAP is based on HMMER gene prediction methods with a sequence similarity-based approach, which combines comparison of the predicted gene products with the non-redundant protein database, Entrez Protein Clusters, the Conserved Domain Database, and the COGs (Clusters of Orthologous Groups). A prerequisite for JCVI tools is a maximum of five contigs/pseudocontigs, which makes them difficult to apply when there are tens of contigs. Artificially-created pseudocontigs formed from too many contigs may add artefacts. For closely-related strains, the transfer of annotation may be the best tool (RATT, Rapid Annotation Transfer Tool [9]), however, in this study we did not use these tools because we found no suitable sibling strains.

The objective of the present study was to test and compare open source short read assemblers with online integrated annotation tools for their potential to automate work flow in *de novo* genomics, with as little human intervention, manual operations, and curation as possible. For *de novo* analysis we used two novel strains from the genus *Flavobacterium* and one strain from the genus *Marinomonas*; for quality control we used a fully re-sequenced organism with a finished genome (*Alcanivorax borkumensis* SK2). Bacteria for *de novo* sequencing originated from the same environment (fresh water rivers and the Northern Baltic Sea [13]), two bacteria are very closely related on the basis of an initial 16S rDNA comparison, while a third bacterium is presumed to possess similar ecophysiological properties.

## Results

### Sequencing output

Genomes were sequenced on two runs using four multiplex identifier (MID) adaptors for each run. Rapid Library (RL) sequencing yielded poor results because of the relatively low read lengths, so the sequencing was repeated using General Library Preparation (GLP). The sequencing outputs are presented in Additional file 1: Table S1. The average read length was shorter using the RL kit than the GLP kit, with fewer total bases and less total coverage. Pooled data with ~ 30 fold coverage from both sequencing runs were used in all downstream data analysis.

### Reference mapping of re-sequencing and annotation/re-annotation

For reference mapping, we used the completed genome of *Alcanivorax borkumensis* SK2 (NC_008260 [gi:19683]) and re-sequenced data from our *A. borkumensis* SK2 strain. Reference mapping did not result in assembly of the full genome. Out of the complete genome (3,120,143 bps), the number of matching bps found depended on the genome assembler used (Table 1). The best performing assembler was MIRA3 which produced 3,119,125 bps, followed by Newbler (3,104,799 bps), and Mosaik (3,099,937 bps). The number of contigs varied depending on the software, from 9 (Newbler) to 18 (Mosaik) with MIRA3 yielding 11 contigs. Manual evaluation of the mapping results of all reads revealed that the most complete coverage was achieved using MIRA3. The break-up of the reference mapping into several contigs happened because seven regions were covered by a few ends of reads with lower quality or by a single highquality read. In addition, three regions with gaps between 41 to 762 bp were not covered by any reads. The overall quality of the MIRA3 mapping was considerably better than that produced by other genome assemblers.

**Table 1:** **Reference mapping results of type strain *Alcanivorax borkumensis *SK2 using pyrosequencing reads and comparison with *de novo *assembly (MIRA3)**

**SOFTWARE**	**Total bps covered by mapping/Length of missing fragments**	**Disagreements**	**Number of bps**
MOSAIK	3 099 937		
	20 206		
		*Ns*	1535
		*Tranversion/Transitions*	2
		*Insertions*	10 225
		*Deletions*	7 733
NEWBLER	3 104 799		
	15 344		
		*Ns*	1 544
		*Tranversion/Transitions*	0
		*Insertions*	11 493
		*Deletion*	10 346
MIRA3	3 119 125		
	1 018		
		*Ns*	905
		*Tranversion/Transitions*	2
		*Insertions*	1 867
		Deletions	5 187
*de novo*	3 079 251		
	40 892		
		*Ns*	323
		*Tranversion/Transitions*	9 814
		*Insertions*	1 491
		Deletions	1 590

The difference between NC_008260 (3,120,143 bps) and the number of nucleotides covered corresponds to the total length of the genome not mapped. Disagreements are indicated as conflicting positions in the consensus sequence between reference genome and mapped reads. Ns – number of fully ambiguous nucleotides within mapped regions.

The automated annotation pipelines RAST, IMG, and PGAAP were evaluated with reference-mapped contigs found using the MIRA3 and Mosaik assemblers. These annotations were compared to the original annotation of the *A. bokrumensis* SK2 (NC_008260 genome from GenBank [gi:19683]); in addition, the raw sequence of SK2 was re-annotated with these three annotation pipelines (Additional file 1: Table S2). Re-annotation of the finished genome sequence increased the number of Coding Sequences (CDSs) by about 5–6%, and the number of tRNA and rRNA operons did not change. The length of the CDSs did not change remarkably and the average length of CDSs in the finished genome was 993 ± 666 (mean ± standard deviation, the largest CDS being 10,803 bp), and in the re-annotated genome 934 ± 677 (the largest being 10,677 bp). In contrast, when using truly re-sequenced data, both the MIRA3 and Mosaik assemblers predicted considerably more CDSs. The CDS size clearly decreased using PGAAP annotation, e.g. 11 contigs from MIRA3 mapping resulted in an average CDS of 490 ± 337 (the largest comprised of only 2,925 bp, which is statistically different from the finished genome at p < 0.01 by Tukey pairwise comparison of one-way ANOVA). This occurred because in the reference mapping mode 100% of the consensus contigs contained many artificial indels, and many more artificial stop codons were introduced (data not shown). These artificial indels originated mostly from homopolymeric regions or other sequencing errors of different pyrosequence reads from the same fragment. Different independent reads contained different numbers of homopolymeric nucleotides of high quality, which led to a consensus of less than 100%, because re-mapping algorithms are optimized to discover SNPs. For example, one aconitase A CDS was fragmented into five pieces using both the IMG and RAST annotation pipelines (Additional file 1: Figure S1). PGAAP behaved even worse as it tended to annotate only the longest CDS with the same name/function, so the remaining sequence regions were left unannotated, and therefore the proportion of non-coding parts increased substantially.

**Figure 1:**
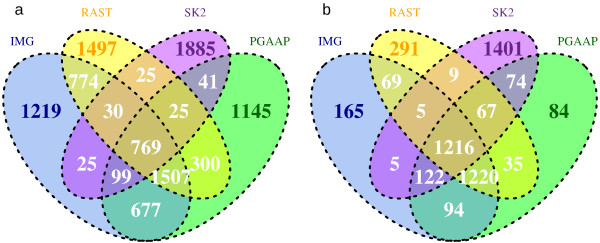
**Venn diagrams of matching (based on 100% similarity) CDS in re-sequenced *A. borkumensis *SK2 genome when annotated by IMG, PGAAP and RAST and compared to re-annotated CDS of *A. borkumensis *SK2 finished genome downloaded as a raw sequence from GenBank. a **– annotations based on genome assembly using reference mapping by MIRA3; **b **– annotations based on de novo genome assembly by MIRA3. Numbers represent count of CDS annotated by different annotation pipelines: IMG, RAST and PGAAP, while SK2 denotes CDS from re-annotation of the NC_008260 in GenBank.

**Figure 2:**
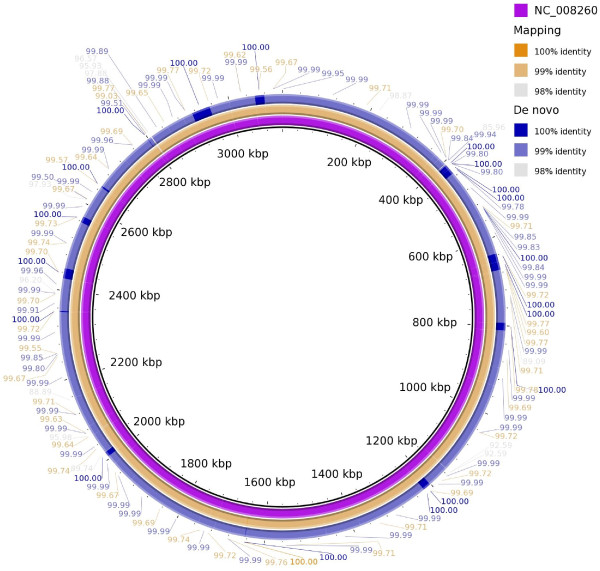
**Comparison between genomes sequences of NC_008260 in GenBank, and genome sequence of re-sequenced strain *A. borkumensis *SK2, which was obtained after reference mapping or after de novo assembly.** Numbered labels indicate exact identity in percentages.

A full comparison of the matching annotation results (CDSs) obtained from the genome assemblers and annotation pipelines used is presented in Figure 1a; CDSs were compared on the basis of sequence match at 100% similarity – to compare CDSs with exact start and stop matches. All pipelines, including the re-annotation pipeline, predicted more CDSs than the original sequencing (NC_008260 in GenBank). Some of these CDSs were unique to each pipeline and were not identified by the others. The number of matches identified increased by 52.8% when re-sequenced and *de novo* assembled data was used compared with the re-annotated sequence data from the finished genome (Figure 1b, 769 and 1507 CDS). This was corroborated by comparing raw sequences; While both the reference mapping and the *de novo* assembly contained sequencing errors (Figure 2), these errors influenced the consensus of reference-mapped reads more severely.

Further manual comparison revealed differences in gene calling that could affect the accuracy and annotation details of genome analyses. The tRNA and rRNA operons were used as examples (Additional file 1: Table S2). Annotation of MIRA3 mapping by RAST was in good agreement with the NC_008260 re-annotation; all three rRNA operons were annotated accurately, and the number of tRNAs was 43 (originally 42). IMG-ER annotation of the same mapping was very similar to RAST: three rRNAs and 43 tRNAs. In contrast, PGAAP failed to find the rRNA operons and only three 5S rRNAs were annotated; the rest of the operon was found in the assembly but was not recognized by PGAAP. Annotation results for tRNAs were similar to RAST and IMG-ER. The greater number of contigs (18) that resulted from Mosaik assembly induced suboptimal results, e.g. RAST failed to annotate any rRNA operons and predicted one fewer tRNA. An exact sequence-based comparison of all three rRNA operons in the re-annotation of NC_008260 and re-sequencing/annotation of our *A. borumensis* SK2 are provided in Additional file 1: Figure S1.

### *De novo* assembly

Three assemblers (MIRA3, Newbler, and CABOG) were compared on the basis of output statistics of the *de novo* assembly using their default parameters in the assembly process (Additional file 1: Table S3). All *de novo* assemblies of the four strains were highly fragmented into relatively small contigs when assembled using CABOG. They contained more contigs in total, and had lower N50 values when compared with the other assemblies. MIRA3 assemblies resulted in longer contigs and higher N50 values when compared with the other assemblies (Additional file 1: Table S3). *De novo* assembly of the re-sequenced genome of *A. borumensis* SK2 using MIRA3 covered 98.7% (3,079,251 of 3,120,143 bps) of the reference genome, which is lower than the reference mapping of the same number of reads. In addition, the pairwise similarity of the reference genome and *de novo* assembly was 95.3% (3,010,624 identical sites). In contrast, *de novo* assembly using CABOG covered only 81.6% of the total genome (2,545,645 of 3,120,143 bps). Newbler covered slightly fewer base pairs than MIRA *de novo* assembly - 98.1% (3,061,666 of 3,120,143 bps).

The genome sizes of novel strains subjected to true *de novo* assembly varied: *Flavobacterium* strain GOBB3-209 had ~2.4 Mbps (GC content: 33.5%) and GOBB3-C103-3 ~4.2 Mbps (34.6 CG%), and *Marinomonas* GOBB3-320 had ~4.8 Mbps (44.6% GC%). The number of assembled contigs for the small genome bacterium (genome ~3 Mbps) was comparable to *A. borkumensis* SK2, which has roughly the same genome size. Therefore, we assume that our *de nov*o assembly might cover ~99% of the total genome of GOBB3-209. Larger genomes (> 4 Mbps) were more fragmented and thus it is difficult to estimate how much of the total genome was covered and annotated using our approach.

### Comparison of *de novo* assembled genomes annotated by automated pipelines

All *de novo* sequenced genomes were annotated using three pipelines: IMG, PGAAP, and RAST. We compared these annotation pipelines using a more robust method. In contrast to the above comparison, we used annotations of protein coding genes that were based on COGs instead of directly comparing the nucleotide sequences of CDSs (Table 2). The COG annotations allowed us to compare the performance of the different pipelines in predicting the general metabolic features of novel organisms – a method predicted to be useful for general genome analysis. At the COG categories level, the numbers of annotations on the same strain were statistically different (pairwise Fisher's exact test, p < 0.05). Using COG annotation, the number of annotations was statistically different in very few cases and occurred only with data from the GOBB3-C103-3 assembly. In general, IMG-ER annotated fewer COGs than either PGAAP or RAST. In addition, the differences and the number of COG annotations missing between comparison annotations were greater for strain GOBB3-C103-3, which has a larger genome and a more fragmented draft assembly (Table 2).

**Table 2:** **Annotation of *de novo *assemblies**

			**IMG-ER**	**PGAAP**	**RAST**
Strain					
SK2	Total COGs		2050	2111	2113
	Missing COGs		116	82	80
	Proportion of missing COGs		8.1	5.8	5.6
	Predicted genome size, Mbs	3.4			
209	Total COGs		1383	1449	1476
	Missing COGs		116	80	66
	Proportion of missing COGs		10.5	7.3	6.0
	Predicted genome size, Mbs	2.4			
C103-3	Total COGs		2123	2212	2256
	Missing COGs		203	154	142
	Proportion of missing COGs		14.8	11.3	10.4
	Predicted genome size; Mbs	4.2			
320	Total COGs		2964	3079	3072
	Missing COGs		132	95	97
	Proportion of missing COGs		7.9	5.7	5.8
	Predicted genome size, Mbs	4.8			

SK2 – *Alcanivorax borkumensis *SK2; 209 – *Flavobacterium* sp. GOBB3-209; C103-3 – *Flavobacterium *sp. GOBB3-C103-3; 320 – *Marinomonas *sp. GOBB3-320. Genome size is given as Newbler default prediction. Missing COGs are COGs that are absent from the annotation compared to NC_008260 annotation in GenBank.

## Discussion

The relative value of draft versus completely finished genomes has been debated for over a decade (e.g. [14]), over which time the finishing of genomes, including bacterial, remains time consuming and costly. However, this is expected to change because techniques to simplify the finishing process are expected to progress in the near future [15]. At the same time, NGS technologies are developing very rapidly in terms of sequence output and reduced cost, which allows genome sequences to be obtained at a reasonable cost. In addition, some NGS technologies can already produce sequence reads comparable in length to traditional Sanger sequences with high coverage (~30 fold) in hours, which should simplify analysis. It is for these reasons that we conducted an update study to assess the usefulness of these data for rapid and preliminary genome studies. This study compares only free and open source tools because these alone open up the possibility for individual researchers and small research groups to both assemble and annotate sequence reads into completed genomes, and tune the analysis pipelines.

Before embarking on bioinformatic genome analysis, researchers are faced with a multitude of choices regarding which analytical protocols and tools to apply – a choice which is especially critical for small and independent research groups. Many bioinformatic tools are either free and open source or freely available for academic use, and are thus natural choices. However, these two types of software differ. Often, the installation, operation, and maintenance of free and open source software requires specific technical expertise and current best practice are either poorly documented or entirely lacking – drawbacks that are expected to decrease rapidly over time. Closed-source tools that limit our academic freedoms, including those without licencing fees, also limit the transparency of their pipelines which may adversely affect the reproducibility of computed results.

Analogous to the choice of analysis tools, the choice of sequencing technology to apply has not been clearly settled. Our results show that Roche 454 pyrosequencing might be a good choice for obtaining *de novo* draft assemblies of environmental bacterial strains relatively economically, however, it remains problematic for re-sequencing projects because of its high error rate. The errors give high quality signals at the single nucleotide level in homopolymeric regions and therefore introduce ambiguities or contradictions into the reference mapping assembly. On the other hand, Roche 454 technology is suitable for *de novo* sequencing and draft assembly, and allows for the automatic annotation of WGS bacterial genomes with relatively low sequencing effort, i.e. currently one or two bacterial genomes per run. Because *de novo* assemblers use a more probabilistic approach, they are less influenced by the erroneous reads of Roche 454 in homopolymeric nucleotide regions. These erroneous nucleotides in individual reads with high quality signals are levelled off by the average frequencies of nucleotide positions, so no artificial indels are introduced and therefore the errors do not disturb the consensus in the same way as in the reference mapping.

Automated annotation of draft genomes provides preliminary information about the genomes of novel organisms and this annotation approach ought not to yield highly erroneous results that may mislead the researcher. On this level, all of the pipelines we tested performed very well; the artefacts in the annotation data mostly originated from the re-mapping assembly and not from the annotation process itself. However, the coding regions were not precisely located (exact start and stop) when compared between different automated annotation tools because none of these tools attempts to locate the origin of replication [16].

### Assembly algorithms

Two algorithms are widely employed in *de novo* genome assembly. The first is the overlap layout consensus (OLC) or overlap contig consensus approach, and the other is the de Bruijn graph (DBG) or Eulerian path [17]. The latter is more useful for shorter reads (<150 bp) numbering hundreds millions compared to a few million 454 Titanium pyrosequencing reads (>400 bp). We used our data to conduct preliminary tests on the effectiveness of Velvet, which implements a DBG-based approach [18], however, the assemblies were highly fragmented (data not shown). For a comprehensive review of *de novo* assembly algorithms we refer the reader to Miller et al. [19].

MIRA3 is a hybrid combination of the OLC and greedy algorithms [20], and is an iterative assembler that learns from past mistakes with OLC and greedy components (B. Chevreux, personal communication). The MIRA3 project is being actively developed and has a growing group of users. MIRA3 performed better in all aspects of bacterial genome assembly using Roche 454 reads and currently seems to have the greatest potential. In addition, it can be combined with high quality reads from Illumina technology into hybrid assembly, which potentially evades bottlenecks in *de novo* genome assembly of relatively simple bacterial genomes and hopefully will allow this step to be fully automated in the near future.

Newbler is a commercial product, and probably uses the OLC approach, developed by Roche Diagnostics, but it is usually freely available to laboratories running Roche 454 sequencing. It is not a free and open source software package, and release descriptions indicate that the originally published algorithm may differ from the current one. In our hands, the available version of Newbler performed almost as well as MIRA3, however, commercial tools cannot be evaluated in full detail. Celera Assembler (CA) is another open source OLC based tool that evolved from a Sanger-era assembler; the revised pipeline for NGS reads including Roche 454 data is generally called CABOG [19].

It should be mentioned that we did not apply paired end (PE) library sequencing and it is clear that such mate pairs would allow more contigs to be closed into scaffolds or at least a list of ordered and orientated longer contigs. We disregarded this kind of approach in the search for the most simple and cost efficient method for assembling bacterial genomes, and in theory a coverage of 30 times should allow an average bacterial genome to be assembled. The bacterial WGS in the contigs we obtained should not lose the correct prediction of too many genes, and from this point of view proper scaffolding is more important for eukaryotic genomes with larger size.

Reference mapping is a different field of genome assembly related to the exercise of alignment; it should be much easier to map NGS reads to very close reference sequences. We used reference mapping only with our test strain and our initial approach was to use only Mosaik [21] for this purpose. Mosaik is a specific reference-guided assembler using the Smith-Waterman algorithm to align a hashed table of short reads to the reference genome. The purpose of this analysis was to compare the finished genome with re-sequenced 454 reads mapped to the reference and to the *de novo* assembly; we did not expect surprises in this process. However, Mosaik was initially unable to close all gaps between the contigs (18 contigs) resulting from re-sequenced data, leaving ~ 20,000 bps out (Table 1). Because the preliminary results were somewhat suboptimal, including several regions that were not covered, we tested the reference mapping performances of Newbler and MIRA3 for comparison. Newbler performed better, but as with the Mosaik mapping it left some regions without mapped reads (overall statistics in Table 1). MIRA3 reference mapping revealed that there were still a few weakly-covered regions although the total coverage (30 fold) should be acceptable. Fine-tuning of the MIRA3 reference mapping would allow all gaps to be closed with coverage of very few reads that were not accepted and thus not mapped by the software.

### Gene prediction and annotation - tools

In most so-called automated online tools, a truly automatic process with no manual intervention remains a design goal of annotation in terms of predicting tRNA, rRNA and protein coding genes – coding sequences (CDSs). The usual pipelines for finding a gene in a raw DNA sequence involve detection of an ORF, finding the gene and predicting its function by comparing it with genes in existing databases (see below). Automated annotators normally use several HMMs (Hidden Markov Models) and BLAST-based gene prediction methods, e.g. tRNAscan-SE [22], and BLASTp and BLASTn for protein coding and RNA genes, respectively.

Thereafter, the CDSs are assigned to annotations based on various functional resources such as COG clusters [23], Pfam [24], TIGRfam, Gene Ontology etc. Functional annotations may be further “grouped” into metabolically relevant “pathways” such as COG functional categories, Entrez Protein Clusters (ProtClustDB [25], FIGfams-subsystems [10], KEGG [26] and MetaCyc [27] pathway collections, etc. Thereafter, annotated genomes might be maintained by integrated network systems such as RAST-SEED, IMG and others. Such networks allow further comparative analysis to be performed easily with no need for the researcher to have deep expertise in bioinformatic algorithms and tools.

All of the annotation pipelines we tested are second generation tools, which try to combine multiple gene-calling algorithms and knowledge databases for comparison with related species and training sets. Therefore, these tools should perform much better than the early gene-prediction methods such as Glimmer [28] and GeneMark [29].

Draft WGS assembly and preliminary annotation using pipelines proved able to describe the core genomes and different metabolic features of novel environmental miroorganisms fairly well: genome size, basic metabolic pathways, the number of tRNAs, but not rRNAs. For example, two *Flavobacterium* strains isolated from the same environment and at the same season showed large differences in genome size; strain GOBB3-209 is 45% smaller than GOBB3-C103-3. Therefore, it is not surprising that strain GOBB3-209 lacks several features found in GOBB3-C103-3, e.g. the capsule and extracellular polysaccharide pathway, the denitrification pathway, etc. (data not shown). Re-annotation of these finished genomes after a re-sequencing project (e.g. *A. borkumensis* SK2 and Roche 454 pyrosequencing in our study) using automated pipelines may be less useful; in our hands, no improvement was observed after re-annotation. For example, even the prediction of rRNA operons was suboptimal; only one copy in all *de novo* sequenced and assembled genomes was located, although most bacteria described so far have more than one rRNA operon [30].

However, it is worrying that most genomes currently deposited in the public database rely on automated methods, because it has been reported that their performance seems not to have improved over several years [16]. The deposited data sets may contain genomes and gene annotations that differ in their degree of precision and resolution owing to the use of different sequencing methods and annotations. The most severe problem is that erroneous and incomplete annotations are often carried over into the public resources and are difficult to trace and correct afterwards. For example, several hundred CDSs might be removed or added and the start sites corrected (e.g. re-annotation of the uropathogenic *Escherichia coli* strain CFT073 [31]), totalling more than 1000 changes when such data are re-evaluated carefully. Even when many biochemical, physiological, and genetic data support broad genome similarity, the current automated annotation tools can fail to predict certain metabolic pathways. Therefore, more detailed studies that combine all types of available data are needed [32].

### Bottlenecks to be considered

The computational efficiency of *de novo* assembly algorithms implemented in free and open source software (e.g. MIRA3) no longer seems to be a bottleneck. At least with longer reads with ~30-fold coverage, a reasonable draft genome can be produced within hours and without manual intervention on direct shotgun sequencing.

On the other hand, the tools supplied by bioinformatic service providers such as RAST, PGAAP, and IMG cannot yet be fully automated and involve manual intervention. It is clear that the performance of these tools will improve as more carefully curated and finished genomes become available to aid in the automation process. However, considering the number of genomes from unique species available today (in the range of several thousand [33]), and because the potential abundance of microbes in nature is huge [34], progress in this area will take time. Nevertheless, the bacterial genomes used in our study are relatively well covered by existing knowledge of phylogenies and databases of fully sequenced and finished genomes. In the GOLD database [35] there are closely-related bacteria from the genus Marinomonas: *M. mediterranea* MMB-1, ATCC 700492 (unpublished), *M. posidonica* IVIA-Po-181 (unpublished) Marinomonas sp. MWYL1 (unpublished) and *Alcanivorax borkumensis* SK (finished) [36]; and from the genus *Flavobacterium*: *F. columnare* ATCC 49512 (unpublished), *F. branchiophilum FL-15*[37], *F. johnsoniae* UW101 [38], and *F. psychrophilum* JIP02/86 [39]. Therefore, after true finishing of the drafts, the genomes of these organisms can be described with no particular effort, after manual curation, at the same level as phylogenetic relatives of finished genomes. Further justification and re-annotation would be based directly on new biological discoveries.

The usefulness of detecting SNPs has been discussed previously [40], and because the error rate of 454 pyrosequencing is higher than Sanger sequencing and probably more than Illumina (Solexa/Genome Analyser), it might be a suboptimal choice for re-sequencing and reference mapping projects. Pop & Salzberg [41] reasoned that fragmented draft genomes would produce fewer annotated genes because of false stop codons; our observations differ but lead to a similar result with regards to reference mapping. The flaws in reference mapping caused by errors introduced by 454 pyrosequencing led to frame shifts and thus to a greater number of CDSs.

We did not use paired-end (PE) libraries because we wanted to keep the preparation cost of sequencing as low as possible. Our results indicate that the PE approach is useful for scaffolding truly *de novo*-sequenced data to a sufficient degree of coverage (~30 fold) even in assembly of relatively small and simple bacterial genomes. Complex genomes containing repetitive elements may need more attention, although the same is true for annotation of genomes containing genomic and/or pathogenic islands. Isolation of genomic DNA by standard methods often fragments the chromosomal DNA into smaller pieces with a size limit of a few tens of kbs, so extended PE libraries with very large fragments are not easy to construct and need specific treatment of the genomic DNA prior to sequencing. Therefore, it is not possible to assemble larger repetitive elements correctly compared to the fragments in DNA extracts.

## Conclusions

*De novo* assembly software and algorithms are powerful enough to allow average bacterial genomes to be assembled within hours or a few days, opening up the possibility for small research groups to study tens or even hundreds of previously unsequenced genomes. Automated annotation allows vast quantities of sequencing results to be processed into meaningful preliminary data, which are useful for general comparison of novel bacterial strains. However, even well-finished bacterial genomes require manual curation and continual updating, especially for key reference genomes. In conclusion, we have to recall a statement made previously [6]: standardization of gene prediction and annotation is of the utmost importance to prevent a heavy burden of incorrect gene calls in genome databases; on the other hand, finer justification of genome annotations is an almost never-ending process, pushed forward mostly by experimental studies (transcriptomics, proteomics, phenotypic tests, etc.).

## Material and methods

### Origins of the strains

Three environmental bacterial strains, *Flavobacterium* sp. GOBB3-C103-3, *Flavobacterium* sp. GOBB3-209 and *Marinomonas* sp. GOBB3-320, were obtained from estuarine enrichment cultures based on samples from a watershed in the northern Baltic Sea [42,43]. The GenBank accession numbers of the 16S rRNA gene sequences were AF321019, AF321038, and AF321017, respectively. One bacterium, type strain *Alcanivorax borkumensis* (strain SK2/ATCC 700651/DSM 11573, TaxID: 393595) [NEWT/NCBI]), was chosen as a reference strain because its genome was fully sequenced and annotated in the pre-NGS era (ref PubMed = 16878126).

### Growth conditions of strains

Strains were isolated from enrichment cultures when the total bacterial abundance reached ~1–2 × 10^6^ cells ml^-1^ (early stationary phase, after approximately two weeks from inoculation). Cultivable bacteria were isolated by spreading 100 μl samples on triplicate ZoBell medium or more nutrient-poor AC medium agar plates with 60 mmol l^-1^ riverine dissolved organic carbon. ZoBell medium [40] was made from 800 ml, 0.2 μm filtered (Durapore, Millipore) seawater and 200 ml of MilliQ ultrapure water (Millipore). This was supplemented with 5 g casein pancreatic digest (Trypton, Difco), 1 g yeast extract (Difco) and 0.01 g FePO_4_.2H_2_O (Aldrich). Plates were incubated in the dark at 15°C until no more colonies appeared (about 5–15 days). Isolates were purified by serially streaking a single cell colony three times on to new agar plates. The purified isolate was grown in ZoBell medium (10 ml) in the dark at 15°C (1–2 days). After the purity of the isolate was confirmed by one more plating it was re-grown in liquid Zobell medium and 0.8 ml of this cell suspension was mixed with 50% glycerol (0.2 ml) and stored at -80°C. Type strain *Alcanivorax borkumensis* SK2 was routinely maintained on Marine Broth 2216 at 30°C, for plating with agar (15 g l^-1^).

### Genomic DNA preparation and quantification, 16S rRNA gene amplification, and sequencing

Genomic DNA was extracted from pelleted pure isolates using a Qiagen DNeasy Blood and Tissue kit (cat. 69504). The amount of genomic DNA was measured using a Qubit Fluorometer (Invitrogen). To check the isolate identity, a partial 16S rDNA gene from the isolated bacteria was amplified and directly sequenced using the Applied Biosystem 3730XL according to the manufacturer’s directions. The bacterial universal primers 27F (3’-AGAGTTTGATCATGGCTCAG-5’) and 1492R (3’-TACGGYTACCTTGTTACGACTT-5’) were used for amplification. The PCR product was purified with PCR Kleen Spin Columns (Bio-Rad Inc) and the nucleotide sequences determined from three partial fragments of the 16S rRNA gene covering almost the full length of the gene. Nucleotide sequences were determined from the purified 16S rRNA gene with the primers 27F (3’-AGAGTTTGATCATGGCTCAG-5’), 800R (3’-CCAGGGTATCTAATCC-5’), 1492R (3’-ACGGGCGGTGTGTRC-5’), and 347F (3’-TACGGGAGGCAGCAG-5’).

### Whole genome sequencing

Purified DNA from each isolate was directly sequenced using Roche standard technology (Roche 454 GS-FLX system, GS Titanium chemistry by Zürich Functional Genomics Centre). The sequencing libraries were prepared with a GS Rapid Library Kit (Roche, cat. no. 05 608 228 001) together with a GS Rapid Library MID Adaptors Kit (Roche, 05 619 211 001) using 500 ng of DNA. In addition, a General Library Preparation Kit (Roche, 05 233 747 001) together with a Titanium Library MID Adaptors kit (Microsynth) was employed using 3.7 μg DNA from *Alcanivorax borkumensis* SK2, 4.5 μg from *Flavobacterium* sp. GOBB3-C103-3 and 5 μg from both *Marinomonas* sp. GOBB3-320 and *Flavobacterium* GOBB3-209, according to the manufacturer's protocol. The sequencing reactions were performed using a Roche 454 Genome Sequencer FLX with the GS Titanium Sequencing Kit XLR70 (Roche, cat. no. 05 233 526 001) using the 2-region gasket of the GS Titanium PicoTiterPlate Kit (70x75) (Roche, cat. no. 05 233 682 001), according to the manufacturer's instructions. Imaging and signal processing were done using GS FLX SW v2.3, gsRunProcessor fullProcessing.

### Analysis of sequencing data – reference and *de novo* assembly and rapid annotations

For reference mapping, Mosaik release 1.0.1388 [18], Newbler Version 2.3 (Roche) and MIRA3 version 3.2.0 [44] were used; raw reads from the Roche sequencing of *Alcanivorax borkumensis* ATCC 700651 DNA isolated in the laboratory and the backbone of *Alcanivorax borkumensis* SK2 (NC_008260 [gi:19683]) were downloaded from NCBI. *De novo* assembly was performed using the Roche reads obtained from all four strains; the assemblers were MIRA3, Newbler v. 2.3 (Roche), and modified Celera Assembler (CABOG Version 6.1, [45]). Multiple contigs obtained from the reference mapping and *de novo* assembly were subjected to three automated annotation tools: RAST [46], NCBI-PGAAP [8], and IMG-ER [7].

## Abbreviations

BLAST: Basic local alignment search tool; CDS: Coding DNA sequence; COG: Clusters of orthologous groups of proteins; DBG: de Bruijn graph; GLP: General library preparation; IMG: Integrated microbial genomes; PGAAP: Prokaryotic genomes automatic annotation pipeline; OLC: Overlap layout consensus; NGS: Next generation sequencing; PE: Paired end; RAST: Rapid annotation using subsystem technology; RL: Rapid library; rRNA: Ribosomal RNA; SAG: Single amplified genome; SNP: Single-nucleotide polymorphism; SRS: Short read sequences; tRNA: Transfer RNA; WGS: Whole genome shotgun.

## Competing interests

The authors declared that they have no competing interests.

## Authors’ contributions

VK's contribution was to build the concept of the study; he designed the acquisition of data, performed all lab work besides Roche sequencing, and conducted the analysis and interpretation of data. He also drafted most of the manuscript. TL was involved in providing the funding, drafting the manuscript and critically revising the final version. Both authors read and approved the final manuscript.

## Supplementary Material

Additional file 1 **Downloadable files with various annotations of rRNA operons from *Alcanivorax borkumensis *SK2. **Files contain the original finished genome annotation on rRNAs, re-annotation of the finished genome sequence by RAST, IMG, and PGAAP, and re-sequenced and re-annotated rRNA operons. Formats used include the raw sequence in FASTA, annotations in GenBank and SAM alignment. Click here for file
