# Supplementary material for: Genome sequencing of bacteria: sequencing, de novo assembly and rapid analysis using open source tools
Source: BMC Genomics. 2013 Apr 1;14:211. doi: 10.1186/1471-2164-14-211 (PMC3618134; doi:10.1186/1471-2164-14-211)

Supplemental DATA

Supplemental File 1. Downloadable alignment file of various rRNA operons from *Alcanivorax borkumensis* SK2 genome. File contains the original finished genome annotation on rRNAs, re-annotation on finished genome sequence by RAST, IMG and PGAAP, and re-sequenced and re-annotated rRNA.

Supplemental Table 1. The raw sequencing output after Roche 454 sequencing when Rapid Library or General Library preparation was used. Genomes are denoted by strain identifier: SK2 – *Alcanivorax borkumensis* SK2; C103-3 – *Flavobacterium* sp. GOBB3-C103-3; 320 – *Marinomonas* sp. GOBB3-320; and 209 – *Flavobacterium* sp GOBB3-209.

	Rapid Library			General Library		
	No. of reads	Bases	Length Average	No. of reads	Bases	Length Average
SK2	193979	45056983	232	195734	58905917	301
C103-3	189321	46026298	243	220154	61492863	279
320	259718	60998349	235	420872	126707997	301
209	333197	80508738	242	280130	81090693	289
Length Average			276.45			333.55
Length Std Deviation			117			128.08
Median Reads Length			272			359
Total Bases		232590368			328197470	

Supplemental Table 2. Finished genome of *Alcanivorax borkumensis* SK2 (NC_008260 in GenBank) compared to re-sequencing of the same strain. In reference mapping either Mosaik or MIRA3 was used. Statistics are given when RAST, PGAAP or IMG were used as annotation tool. CDS – coding sequences, lsu - large subunit rRNA, ssu – small subunit rRNA, tsu – 5S subunit rRNA.

			Numbers of			
	Annotation tool	Total length, bp	Number of contigs	CDS	tRNAs	rRNA operons
Finished genome						
NC_008260	original*	3 120 143	1	2755	42	3
	RAST**			2910	42	3
	PGAAP**			2901	42	3
Reference mapping						
Mosaik	RAST	3 102 433	18	6877	41	none
	PGAAP			4638	42	3 copies of tsu + ssu, lsu fragments***
	IMG			6422	41	fragments***
MIRA3	RAST	3 116 196	11	4878	43	3
	PGAAP			3745	43	3 copies of tsu****
	IMG			5045	43	3
De novo assembly						
MIRA3	RAST	3 114 849	27	2925	40	1 full operon, 5 fragmental rRNAs
	PGAAP			2862	40	3 copies of tsu****
	IMG			2837	40	1 full operon, 8 fragmental rRNAs

*- original annotation from [29] accessed from Genbank; ** - re-annotation on the finished genome using automated pipelines (RAST and PGAAP); *** - fragmental prediction only; **** - all three TSUs annotated correctly, and some fragments of ssu/lsu; ***** - annotated only tsu's, failed in annotating any ssu and lsu.

Supplemental Table 3. Raw statistical comparison of *de novo* genome assemblies by MIRA3, Newbler or CABOG. SK2 – *Alcanivorax borkumensis* SK2; C103-3 – *Flavobacterium* sp. GOBB3-C103-3; 320 – *Marinomonas* sp. GOBB3-320; and 209 – *Flavobacterium* sp GOBB3-209.

N50 - is defined as the length contigs (N) for which 50% of all bases in the assembly are in a sequence of length L < N.

**Strain**	**SK2**			**209**			**C103-3**			**320**		
Assembler	*MIRA3*	*Newbler*	*CABOG*	*MIRA3*	*Newbler*	*CABOG*	*MIRA3*	*Newbler*	*CABOG*	*MIRA3*	*Newbler*	*CABOG*
Assembled bps	99 889 659	103 369 499	91 484 790	154 409 472	160 627 161	155 985 077	144 093 063	150 237 062	144 966 233	180 080 820	186 389 527	181 570 679
Average recovery	32	31	29	64	66	44	33	35	28	37	39	34
Size of consensus	3 114 849	3 100 791	3 155 926	2 301 590	2 290 996	864 250	4 200 016	4 115 993	3 479 714	4 511 028	4 447 860	4 434 168
Nr of large contigs (>500bp)	31	35	670	37	56	595	108	150	1612	81	120	1422
Largest contigi	293 122	291 961	23 122	350 125	236 138	4 176	224 076	197 209	21 222	507 454	255 787	16 870
N50	214 864	181 385	6 311	103 296	78 664	1 448	70 240	57 040	2 346	100 024	91 292	3 832

Supplemental Figure 1. Introduction of artificial stop codons into reference mapped annotation of *A. borkumensis* SK2. As an example, the aconitase A CDS is broken into several CDSs. A. Comparison between NC_008260 in GenBank with re-sequenced and mapped assembly. B. Artificial deletion is introduced into the homopolymeric region because there is no 100% consensus on reads of one nucleotide. C. Difference between single reads from “problematic” region.

Supplemental Figure 1

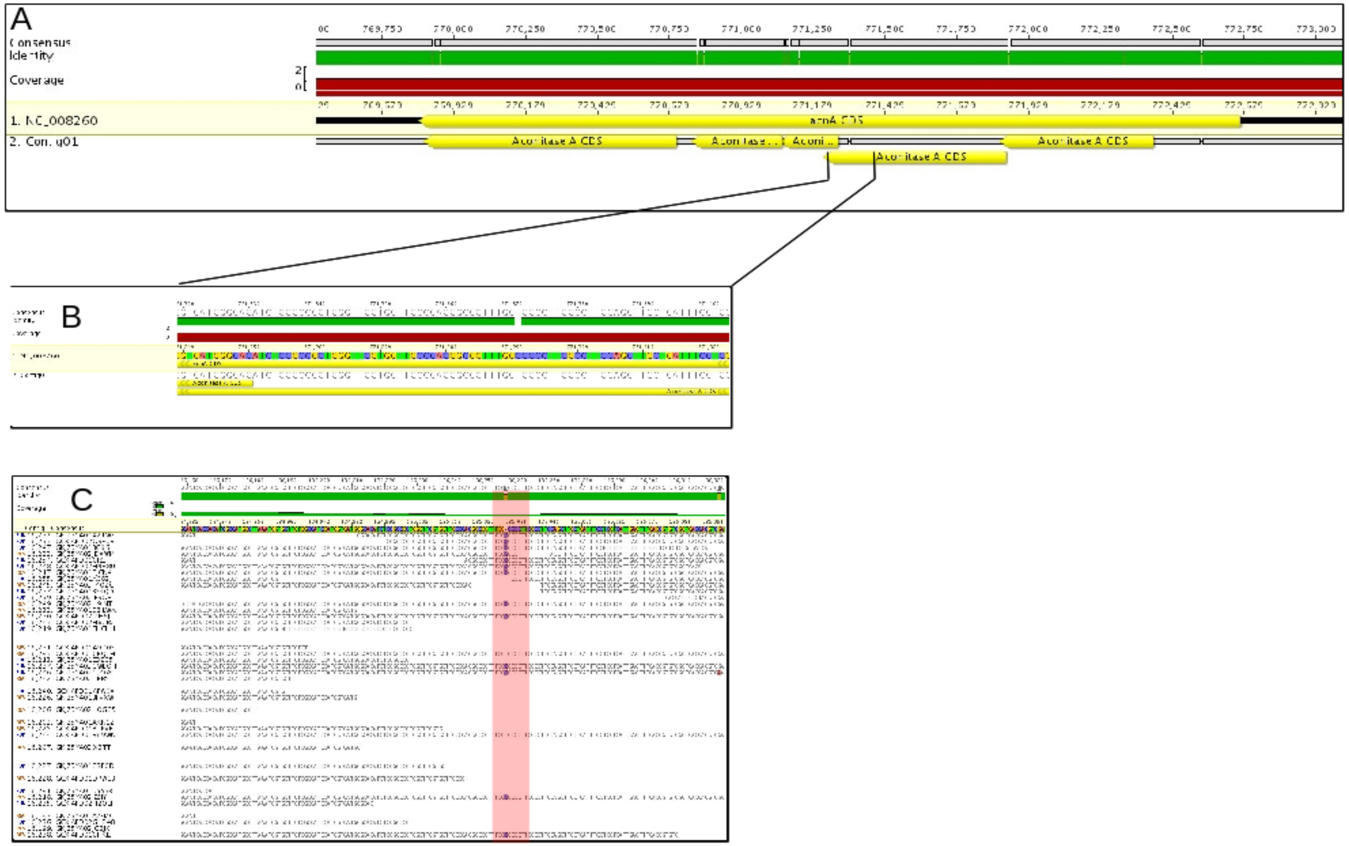

Supplement: Additional file 1 — Downloadable files with various annotations of rRNA operons from Alcanivorax borkumensis SK2. Files contain the original finished genome annotation on rRNAs, re-annotation of the finished genome sequence by RAST, IMG, and PGAAP, and re-sequenced and re-annotated rRNA operons. Formats used include the raw sequence in FASTA, annotations in GenBank and SAM alignment. [file 1471-2164-14-211-S1.pdf]
